# Medical Education in New Orleans

**Published:** 1859-09

**Authors:** 


*Medical Education in New Orleans.*—The editor of the *Medical News and Hospital Gazette*, of New Orleans, in the August number of that journal, states that the city continues remarkably healthy, and that there is every reason to think it will escape this year a visitation of the yellow fever. He adds, "This being the case, we shall expect to see in New Orleans over six hundred medical students; and, in a few more years, our schools will rival in numbers those of Philadelphia, as they now surpass them in clinical advantages."

